# Enhancing critical care practitioners’ knowledge and adherence to ventilator-associated events bundle: a comprehensive analysis

**DOI:** 10.3389/fmed.2024.1365742

**Published:** 2024-11-20

**Authors:** Samiyah Alanazi, Wadi B. Alonazi

**Affiliations:** Department of Health Administration, College of Business Administration, King Saud University Riyadh, Riyadh, Saudi Arabia

**Keywords:** cross-sectional survey, critical care units, healthcare professionals, ventilator-associated events bundle, compliance and knowledge, preventive measures and practice

## Abstract

**Objectives:**

Few surveys have focused on ventilator-associated pneumonia occurring in critically ill patients undergoing intubation and mechanical ventilation. Limited knowledge among healthcare workers may impede compliance with evidence-based guidelines for preventing ventilator-associated pneumonia. We evaluate the knowledge of intensive care professionals related to preventing ventilator-associated pneumonia in the intensive care units.

**Design:**

Cross-sectional survey.

**Setting:**

Adult critical care departments in four tertiary hospitals in Riyadh in Saudi Arabia.

**Subjects:**

Adult intensive care units attending physicians (intensivist, non-intensivist), Nurses, and Respiratory Therapist who works in ICUs.

**Measurement and main results:**

We analyzed 758 questionnaires (100% response rate) from four tertiary hospitals in Riyadh provinces. Nurses constitute the largest group, with most of all professions being Saudi nationals at [343(45.3%)]. Physicians are primarily male, accounting for [127(16.8%)], while the Respiratory Therapy field is predominantly female at [91(12%)]. Our analysis involved, chi-square test to explore potential variations in knowledge among participants with diverse demographic variables. The finding of this was significant positive correlation between some elements. It provides valuable insights into the intricate associations between demographic characteristics and healthcare practices related to VAP prevention (*p* < 0.05). Demographic factors significantly influence health practices related to ventilator associated pneumonia bundle prevention.

**Conclusion:**

Our research identifies key factors influencing ventilator associated pneumonia prevention in critical care settings and provides actionable recommendations for healthcare institutions to enhance patient safety. While this research has extensively examined physicians, nurses and respiratory therapists, there is a depth of investigations comparing the knowledge and practices of these specialists within tertiary hospitals in Riyadh. Conducting such a study is imperative to address knowledge gaps and promote practices that mitigate the adverse outcomes of ventilator associated pneumonia on healthcare systems. This study underscores the pivotal role of education, professional experience, and demographic factors in shaping medical procedures and practices. Targeted interventions in these areas could potentially enhance adherence to the bundle. The study suggests the importance of targeted education programs, mentorship initiatives, and ongoing research to enhance patient outcomes in critical care settings.

## Background

Multiple organ failure often involves major organs, including the lungs. Therefore, it is crucial to provide appropriate ventilation with minimal complications. One of the leading causes of death from hospital-acquired infections is nosocomial pneumonia, which is an infection that a patient acquires during a hospital stay. This type of pneumonia has a crude mortality rate of approximately 30% (1). A specific type of nosocomial pneumonia is ventilator associated pneumonia (VAP). VAP is a bacterial pneumonia that develops in patients who are receiving mechanical ventilation. According to the Centers for Disease Control and Prevention (CDC) in 2023, it is essential for healthcare providers to base their practice on a solid foundation of scientific knowledge to ensure high-quality patient care (2). One way to achieve this is by following evidence-based guidelines for preventing VAP, which can improve patient outcomes (3). Improved outcomes can lead to shorter stays in the Intensive Care Unit (ICU) and hospital, reducing hospital costs for patients. Hospitals also benefit by providing cost-effective services to their patients and communities (4).

Ventilator-associated pneumonia (VAP) bundles of care” is suggested by the Institute of Healthcare Improvement (IHI) for decreasing morbidity and mortality among patients with VAP (5). These bundle components include 30–45° head elevation, closed suctioning and subglottic drainage, daily extubation readiness assessment, deep venous thrombosis (DVT) prophylaxis and oral care. Subglottic drainage is a technique that removes secretions from around the vocal cord and trachea to prevent them from entering the lungs. This program has saved over 122,000 lives, reduced length of mechanical ventilation, and shortened hospital stays according to IHI (6). However, in order to successfully eliminate cases of VAP, compliance rates with the VAP bundle need to exceed 95% (7). It is advisable for long-term adherence for medical as well as nursing staffs’ performance measurement systems to be done on a regular basis (8). There are numerous studies indicating that implementation of these preventive measures greatly reduces VAP rates thus improving patient safety and quality care. Several organizations, including the European Respiratory Society, the Society for Healthcare Epidemiology of America, the Intensive Care Society, the American Thoracic Society, the Center for Disease Control and Prevention, and the Institute for Health Care Improvement, have introduced clinical practice guidelines to improve the prevention of VAP. These guidelines include maintaining oropharyngeal hygiene, suctioning endotracheal secretions, elevating the head of the bed at an angle of 30–45°, providing oral care with chlorhexidine, interrupting sedation daily, utilizing subglottic secretion drainage, practicing proper hand hygiene, monitoring the cuff pressure of the endotracheal tube, and promoting early mobilization (5, 9–14). Despite its use, VAP remains the most common hospital-acquired infection in intensive care units (ICUs), with rates as high as 7.92 per 1,000 ventilator days in 2017 (15).

Timely and suitable treatment has consistently lowered death rates. However, the effectiveness of such treatments is often compromised by the existence of multi-drug-resistant pathogens, both Gram-negative and Gram-positive. Besides the act of endotracheal intubation itself, other factors contributing to VAP include serious underlying conditions (like coma, acute lung injury, aspiration gastric colonization) and various interventions (such as H2 blockers medication, reintubation, supine head position, low endotracheal tube cuff pressure). This knowledge was utilized in the early 2000s to create “ventilator bundles,” which significantly decreased the reported incidents of VAP. There was even a time when it was thought possible to achieve “zero VAP,” with the assumption that VAP was a medical error that could be completely avoided through simple measures like elevating the head of the bed, daily awakening and weaning, and providing oral care (1, 16, 17).

VAP remains a common and potentially fatal complication in ICUs for patients receiving mechanical ventilation. Critical care nurses face the challenge of incorporating evidence-based practices to deliver high-quality care (18). The bundled practices approach consists of individual preventive measures to reduce the incidence and prevalence of VAP and improve patient outcomes. Ali conducted a study in 2013, to assess critical care nurses’ knowledge and compliance with the VAP bundle. The study recruited 45 critical care nurses from different critical care units at Cairo University Hospital. Data were collected between March 2010 and September 2011, including a questionnaire on knowledge and direct observation of nurses providing care to mechanically ventilated patients using a VAP bundle compliance checklist. The results showed unsatisfactory knowledge scores and low compliance with VAP bundle practices among the nurses. The study recommended developing and implementing a protocol for VAP prevention in ICUs, as well as training programs for nurses on infection control and VAP bundle preventive measures to reduce the prevalence of VAP (19, 20).

In 2021, a study was conducted to assess the knowledge, practices, and adherence of nurses and Infection Control Preventionists (ICPs) to the VAP bundles of care in the Intensive Care Unit (ICU). The study involved 60 participants (56 nurses and 4 ICPs) and used qualitative and quantitative tools. The average knowledge score regarding specific evidence-based VAP guidelines was 5 out of 10 points. Self-reported adherence to the VAP bundle ranged from 38.5 to 100%, with perfect compliance in elevating the head of the bed and the poorest compliance in readiness to extubate. The study revealed a lack of knowledge regarding specific components of VAP prevention. It recommended regular formal training and interactive educational sessions to assess the competency of key personnel in implementing the VAP bundle, especially considering the rapid turnover of nurses. Additionally, incentives for nurse retention should be considered to enhance knowledge of hospital-specific initiatives such as the VAP bundles of care over the course of time (21).

In a study conducted by Al-Sayaghi (22), focused on understanding the adherence of critical care nurses to guidelines for preventing ventilator-associated pneumonia (VAP), as well as identifying the factors that influence this adherence. The study also aimed to uncover the challenges nurses face when implementing these guidelines. The research was carried out as a cross-sectional descriptive survey, utilizing a self-administered questionnaire that was distributed to critical care nurses in adult ICUs in Almadinah Almunawwarah, KSA. The questionnaire encompassed recommended strategies for VAP prevention and potential obstacles. Out of the 283 nurses who were invited to participate in the study, 229 responded. The average compliance score was found to be 85.9%, with more than half of the participants demonstrating high or acceptable levels of compliance. The least compliance was observed in the suctioning of subglottic secretions. The main barriers reported by the nurses included a lack of sufficient nursing staff, forgetfulness, and hospital cost control policies. It was also found that working in general ICUs with a capacity of 10–15 beds or having prior education related to VAP prevention had a positive impact on the nurses’ compliance. The study found that the compliance of critical care nurses with VAP prevention guidelines was generally acceptable. However, it also underscored the need to address certain barriers, such as staffing shortages, forgetfulness, and cost control policies, to further improve compliance (22).

Humayun et al. (23) and others conducted a study to determine the prevalence of ventilator associated pneumonia in Saudi Arabia’s healthcare system. Conducted across a wide range of ICUs in Ministry of Health (MOH) hospitals, the study not only quantifies the rates of VAP and ventilator utilization ratios but also compares them with international standards. The findings of this study are particularly important as they provide a unique national benchmark for VAP. This benchmark can serve as a reference point for hospitals across the country, fostering a culture of competitiveness and encouraging continuous improvement in healthcare practices. The study underscores the importance of monitoring and managing VAP rates in ICUs, given the serious implications of this condition for patient health outcomes. Out of numerous ICUs invited to participate, a considerable number responded. The mean VAP rate was compared with international standards, with a sizable portion of the sample demonstrating high or acceptable levels. The lowest rates were observed in areas with high VAP rates. The primary reported challenges were monitoring and managing VAP rates in ICUs, given the serious implications of this condition for patient health outcomes. Working in ICUs with a high VAP rate or having prior education related to VAP prevention influenced the rates. Overall, the rates of ventilator associated pneumonia in Saudi Arabia’s healthcare system were deemed acceptable. The study highlighted the need to address challenges such as continuous monitoring, timely intervention, and adherence to best practices in managing VAP in ICUs to improve patient care (23).

Therefore, this study aims to assess critical care healthcare professionals’ knowledge with ventilator-associated pneumonia bundle at the adult critical care department tertiary hospitals in Riyadh.

## Materials and methods

### Study design

This is a cross-sectional survey study conducted in accordance with ethical guidelines for medical research. All participants provided informed consent, and the confidentiality of all participants was maintained.

### Sampling frame

This study was conducted at an adult critical care departments (CCD) in four tertiary hospitals in Saudi Arabia. These hospitals have 389 ICU beds in total, whereas equipped with advanced medical technology and staffed by a team of highly trained healthcare professionals, including doctors, nurses, and respiratory therapists. The environment in the ICU is often fast-paced and high-stress, as the team works around the clock to monitor and treat patients.

### Study population

The study was conducted at adult critical care departments (CCDs) in four tertiary hospitals in Riyadh, Saudi Arabia. These hospitals have a total of 389 ICU beds, equipped with advanced medical technology and staffed by a team of highly trained healthcare professionals, including doctors, nurses, and respiratory therapists. The environment in the ICU is often fast-paced and high-stress, as the team works around the clock to monitor and treat patients. The subjects included physicians, nurses, and respiratory therapists (RTs) working in CCDs. Utilizing a sample size calculator from Raosoft (24), based on the estimated manpower workforce of these hospitals and a margin of error of 5%, a confidence level of 95%, a population size of 500,000, and a response distribution of 50%, the recommended sample size for the survey was 384 participants to ensure reliable data collection. We successfully obtained 758 completed questionnaires, exceeding the recommended sample size.

### Questionnaire development and testing

The questionnaire was piloted among 15 MPs, and they were excluded from the final analysis. The test was used before in previous study and the reliability of the questionnaire was tested (*r* = 0.962) using Cronbach’s alpha (25).

The data was collected using a structured questionnaire consisting of two parts: The first part of the questionnaire included baseline information, Socio-demographics like: age, gender, the highest educational qualification, professional experience, nationality, and information about evidence-based training taken on prevention of VAP. The second part: The tool included 13 multiple-choice questions regarding the aspects of the prevention of VAP. All the questions have four options, of which three were incorrect and one was correct. The structured knowledge questionnaire was validated by medical experts and scored as either one point for a correct response or zero points for an incorrect response.

### Questionnaire administration

#### Sampling technique

The subjects were invited through a convenience sampling, including physicians, nurses, and respiratory therapists (RTs) working in CCD.

#### Data collection instrument

The Survey was collected using a structured questionnaire. The participants were invited to be part of it through sending it to their work email or their personal phone number.

#### Questionnaire administration

The participants were invited to take part in the survey through convenience sampling. The structured questionnaire was distributed through multiple channels, including work emails, personal phone numbers, and social media apps like WhatsApp.

### Statistical analysis

The primary variables of interest were the knowledge of the practitioners to the VAP bundle. The knowledge was measured based on the survey responses. The collected data underwent comprehensive analysis using the Statistical Package for the Social Sciences (SPSS). Descriptive statistics, including means, percentages, and standard deviations, provided a comprehensive overview of the dataset. Comparative analyses, such as chi-square tests, inquired into potential differences in knowledge and adherence patterns across the diverse specialties of the participants. *p* < 0.05 was considered statistically significant.

## Results

Table 1 provides a comprehensive analysis of demographic characteristics across three healthcare professions: Physicians, Nurses, and Respiratory Therapists (RTs). Nurses constitute the largest group, with the majority of all professions being Saudi nationals at 45.3% (*n* = 343). Physicians are primarily male, accounting for 16.8% (*n* = 127), while the Respiratory Therapy field is predominantly female at 12% (*n* = 91). In terms of education, a significant number of nurses, 25.1% (*n* = 190), hold bachelor’s degrees. Experience levels vary, with Nurses exhibiting a range of experience levels and the majority of all professions having between 4 and 6 years of experience, except for RTs. A noteworthy percentage of all professions have received training on Evidence-Based Guidelines for ventilator associated pneumonia (VAP) prevention, indicating a commitment to continuous professional development.

**Table 1:** Characteristics among three healthcare professions works in critical care units in tertiary hospitals in Riyadh (*n* = 758).

Demographic characteristics of the sample		Physician		Nurse		RT	
		*n*	%	*n*	%	*n*	%
Gender	Male	127	16.8%	223	29.4%	72	9.5%
	Female	53	7.0%	192	25.3%	91	12.0%
Age	20–30 years	51	6.7%	34	4.5%	4	0.5%
	31–40 years	68	9.0%	178	23.5%	25	3.3%
	41–50 years	56	7.4%	191	25.2%	85	11.2%
	More than 50 years	5	0.7%	12	1.6%	49	6.5%
Education	Diploma degree	46	6.1%	39	5.1%	3	0.4%
	Bachelor’s degree.	86	11.3%	190	25.1%	29	3.8%
	Master’s degree.	38	5.0%	178	23.5%	90	11.9%
	PhD or Doctorate degree.	10	1.3%	8	1.1%	41	5.4%
Experience	< I	33	4.4%	12	1.6%	1	0.1%
	1–3 years	63	8.3%	96	12.7%	7	0.9%
	4–6	66	8.7%	216	28.5%	62	8.2%
	7–9	13	1.7%	64	8.4%	70	9.2%
	More than 10	5	0.7%	27	3.6%	23	3.0%
Nationality	Saudi	160	21.1%	343	45.3%	153	20.2%
	Non-Saudi	20	2.6%	72	9.5%	10	1.3%
Have you ever had training or educational courses about evidence-based guidelines (EBGs) on VAP prevention within your employment period?	Yes	165	21.8%	392	51.7%	155	20.4%
	No	15	2.0%	23	3.0%	8	1.1%

**Figure 1:**
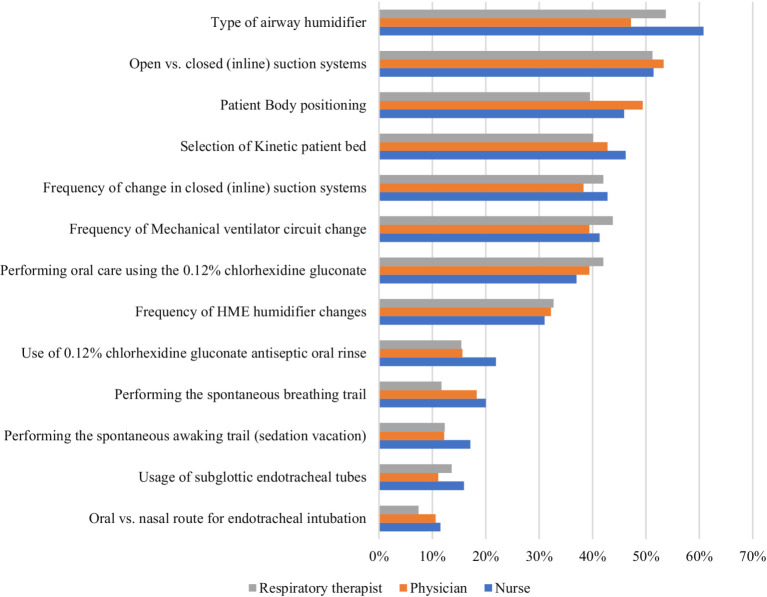
Survey of preventive practices for ventilator-associated pneumonia by healthcare role in Riyadh (*n* = 758).

In Figure 1, we present the outcomes of an extensive 13-question multiple-choice survey meticulously crafted to evaluate the knowledge of healthcare providers—encompassing nurses, physicians, and respiratory therapists (RT) regarding VAP. The questions were strategically formulated to encompass a wide range of topics related to VAP, with the intention of offering a comprehensive overview of healthcare providers’ awareness and proficiency in these critical domains. The questions featured a single correct answer, and the highest rate of correct responses was observed within the nursing specialty. The insights garnered from these responses shed light on the current landscape of VAP knowledge among healthcare providers, pinpointing areas where additional education or training might be beneficial. The responses unveiled a diverse array of preferences and practices across these professional domains, underscoring the intricate and individualized nature of healthcare settings. For example, concerning the preferred route for endotracheal intubation, 11.50% of nurses, 10.60% of physicians, and 7.40% of respiratory therapists opted for the oral route. In contrast, consensus was more pronounced regarding the type of airway humidifier, with 60.80% of nurses, 47.20% of physicians, and 53.70% of respiratory therapists advocating for the same frequency. Notably, the use of 0.12% chlorhexidine gluconate antiseptic oral rinse was less prevalent across all professions, with only 21.90% of nurses, 15.60% of physicians, and 15.40% of respiratory therapists reporting its implementation. These findings accentuate the diversity inherent in practices and preferences within healthcare settings, emphasizing the ongoing need for education, and research to ensure optimal patient outcomes. Furthermore, they lay a solid groundwork for future studies aimed at probing the underlying reasons behind these preferences and practices, as well as their tangible impact on patient outcomes.

Table 2 presents the outcomes of Chi-square tests that explored the correlations between various characteristics (Gender, Job Description, Age, Education, Professional Experience, Nationality, and Training Course) and 13 survey questions, each with one correct answer and three incorrect answers. This analysis provides insights into how these demographic variables relate to specific patient care practices.

**Table 2:** Thirteen multiple choices VAP bundle questions with one correct answer correlated with seven characteristics (*N* = 758).

Ch	Oral vs. nasal route for endotracheal intubation	Frequency of mechanical ventilator circuit change	Type of airway humidifier	Frequency of HME humidifier changes	Open vs. closed (inline) suction systems	Frequency of change in closed (inline) suction systems	Usage of subglottic endotracheal tubes	Selection of kinetic patient bed	Patient body positioning	Use of 0.12% chlorhexidine gluconate antiseptic oral rinse	Performing oral care using the 0.12% chlorhexidine gluconate	Performing the spontaneous awaking trail (sedation vacation)	Performing the spontaneous breathing trail
Sex	= 0.34	= 0.93	= 0.36	= 0.92	= 0.4	= 0.26	**= 0.03**	**≤ 0.01**	= 0.83	**≤ 0.01**	= 0.9	= 0.07	= 0.05
Role	= 0.34	**= 0.01**	= 0.71	= 0.91	= 0.9	= 0.6	= 0.30	=0.31	= 0.17	= 0.08	= 0.53	= 0.18	= 0.07
Age	**≤ 0.01**	= 0.36	**≤ 0.01**	= 0.67	= 0.57	**= 0.02**	**= 0.04**	= 0.9	**≤ 0.01**	**= 0.04**	**= 0.03**	≤ 0.01	**≤ 0.01**
Ed.**	**≤ 0.01**	= 0.34	**= 0.01**	= 0.13	= 0.45	**= 0.05**	**≤ 0.01**	= 0.9	**≤ 0.01**	**= 0.03**	= 0.08	**≤ 0.01**	**≤ 0.01**
Exp.**	**≤ 0.01**	= 0.86	**= 0.01**	**≤ 0.01**	**= 0.01**	**= 0.01**	**≤ 0.01**	**≤ 0.01**	**≤ 0.01**	**≤ 0.01**	= 0.07	**≤ 0.01**	**≤ 0.01**
Nationality	**≤ 0.01**	= 0.8	= 0.63	= 0.06	= 0.08	**= 0.04**	**≤ 0.01**	**≤ 0.01**	**≤ 0.01**	**≤ 0.01**	**≤ 0.01**	**≤ 0.01**	**≤ 0.01**
Training	**≤ 0.01**	**= 0.02**	**= 0.14**	= 0.40	= 0.73	**= 0.01**	**≤ 0.01**	= 0.40	**≤ 0.01**	**≤ 0.01**	**≤ 0.01**	**≤ 0.01**	**≤ 0.01**

Bold values indicate *p*-values; *p < 0.05 are considered statistically significant. **Ed. = Education; **Exp. = Professional Experience.

### Gender

Gender exhibited significant associations with practices such as the selection of kinetic patient beds (*p* = 0.005) and the application of 0.12% chlorhexidine gluconate antiseptic oral rinse (*p* = 0.001). These results suggest that gender differences may influence the choice of certain patient care interventions. For instance, the significant association with the selection of kinetic patient beds indicates that gender may impact decisions related to patient mobility and comfort.

### Job description

Job description was significantly associated with only two practices: the frequency of mechanical ventilator circuit changes and the oral vs. nasal route for endotracheal intubation. This indicates that the role or responsibilities of healthcare professionals could influence these specific practices, but not others.

### Age

Age showed significant associations with multiple practices, including the oral vs. nasal route for endotracheal intubation (*p* < 0.001) and the type of airway humidifier (*p* < 0.001). This suggests that age may affect decisions related to patient airway management and equipment choices, potentially reflecting variations in experience or familiarity with different practices.

### Education qualifications

Education qualifications were significantly associated with practices such as the oral vs. nasal route for endotracheal intubation (*p* < 0.001) and the type of airway humidifier (*p* = 0.014). This implies that educational background may impact knowledge and practices related to patient care techniques and equipment.

### Professional experience

Professional experience demonstrated significant associations with nearly all practices, except for the frequency of mechanical ventilator circuit changes and the type of airway humidifier. This broad impact highlights the influence of professional experience on various aspects of patient care, suggesting that more experienced practitioners may follow different practices compared to those with less experience.

### Nationality

Nationality was significantly associated with practices such as the oral vs. nasal route for endotracheal intubation (*p* < 0.001) and the usage of subglottic endotracheal tubes (*p* < 0.001). This indicates that nationality may influence certain practices, possibly reflecting regional or cultural differences in patient care approaches.

### EBP training course

EBP training course also showed significant associations with practices including the oral vs. nasal route for endotracheal intubation (*p* < 0.001) and the usage of subglottic endotracheal tubes (p < 0.001). This suggests that participation in Evidence-Based Practice (EBP) training courses has a substantial impact on specific patient care practices, indicating a positive effect of training on clinical decision-making.

**Figure 2:**
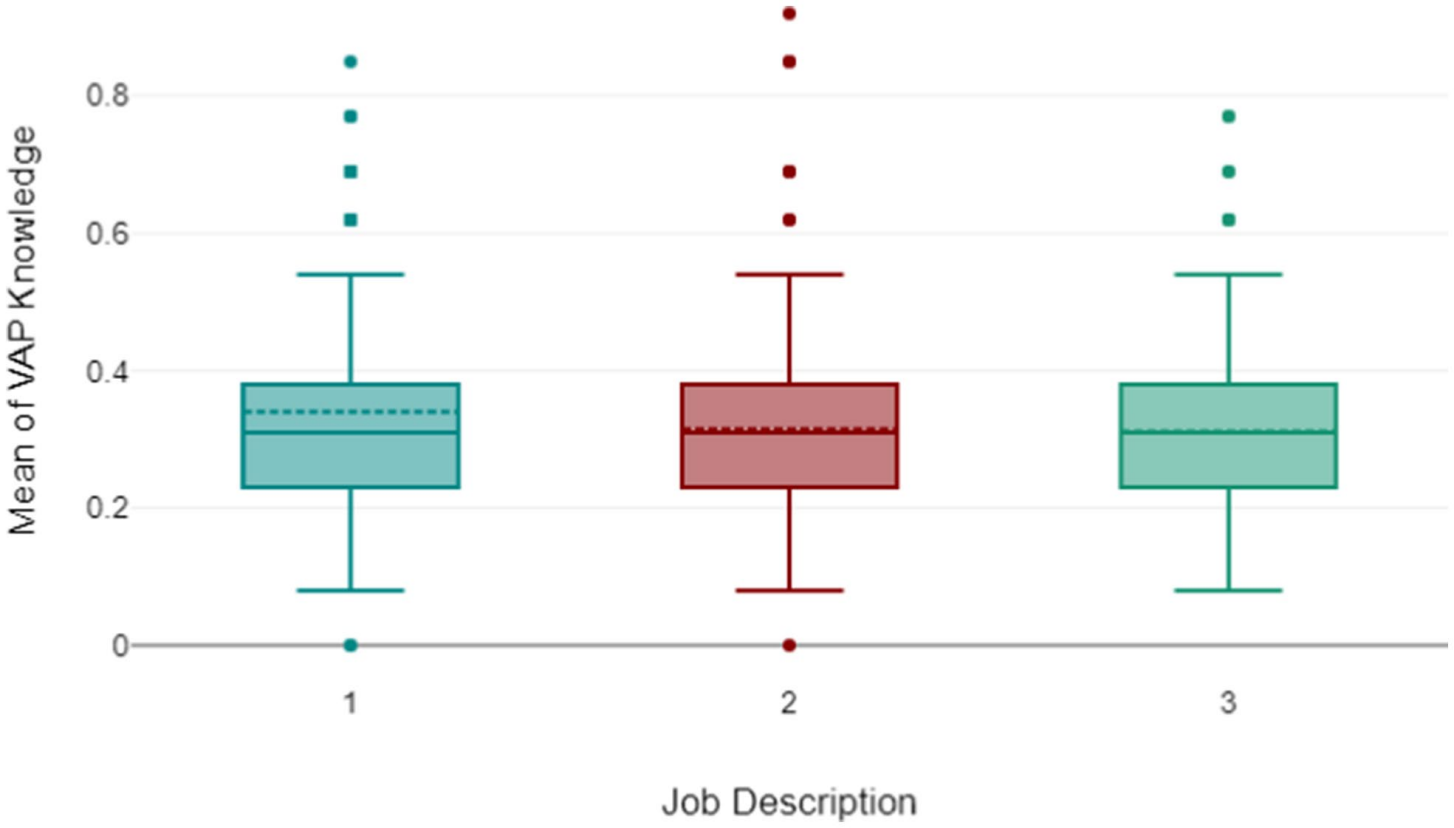
Comparison of adherence levels among nurses, physicians, and respiratory therapists.

In summary, the Chi-square test results reveal significant associations between demographic variables and patient care practices. Notably, professional experience, nationality, and EBP education courses were associated with nearly all bundle elements, indicating their strong influence on practice. Age and education also showed significant associations with several practices. In contrast, gender and job description were associated with only a few practices. These findings highlight key factors that affect patient care practices and underscore the need for further investigation into their implications for healthcare delivery (Figure 2).

In Figure 2, the box plot analysis of three healthcare specialties as one for Nursing, two for Physician, and three for Respiratory Therapist (RT) - in relation to the mean scores for correctly answered VAP questions, revealed noteworthy patterns. All three groups exhibited an equal median score, suggesting a comparable level of VAP knowledge across the specialties. The Nursing group, despite its little higher mean scores, demonstrated a consistent understanding of VAP. The physician and the respiratory therapist group, while having a slightly lower mean score than the nurses, displayed a narrow range of scores, indicating a more uniform level of knowledge within these groups. This suggests a greater scope for improvement in VAP knowledge within these groups. However, the presence of several outliers in these groups points to significant variations in individual performance.

In summary, while nursing group appears to have the highest average VAP knowledge, there is considerable variation within each specialty. The outliers could be attributed to factors such as individual experience, training, or interest in VAP bundle. These findings underscore the potential benefits of targeted training or resource allocation to support those who are underperforming, as well as the opportunity to learn from high performers. This analysis provides valuable insights for enhancing VAP knowledge across all specialties.

## Discussion

The primary objective of this study was to evaluate the comprehension and adherence of critical care health professionals to the ventilator-associated pneumonia bundle within adult critical care department in tertiary hospitals in Riyadh. The investigation stemmed from various influential factors necessitating a comprehensive exploration of healthcare professionals’ understanding of this critical bundle. The survey comprised two main sections: demographic information and knowledge and compliance of practitioners with the VAP bundle.

### Gender disparities in knowledge

Our analysis initially indicates that gender is not significantly correlated with the mean levels of adherence and knowledge. However, upon a more detailed examination of the survey questions, we uncovered significant associations between gender and specific practices. Notably, the selection of kinetic patient beds (*p* < 0.05), and the performance of oral care using 0.12% chlorhexidine gluconate (*p* < 0.05). Compelling, our findings revealed that females exhibited lower knowledge levels compared to males. This contradicts the results of a study conducted in Sana’a, Yemen by Al-Jaradi (26). Which emphasized higher knowledge among females. This contrast prompts the need for further investigation into potential contextual and cultural influences (26).

### Job description and ventilator-related practices

The multifaceted nature of these associations warrants in-depth investigation. Attractively, the frequency of mechanical ventilator circuit change exhibited a significant association with job description, suggesting that distinct roles within the healthcare setting may influence ventilator-related practices. Although, the Job differences and adherence of the VAP mean were not significantly correlated (*r* = 0.014, *p* = 0.694) based on our analysis. This underscores the need for a further exploration of professional roles and responsibilities in shaping adherence to VAP prevention protocols. And it shows that there is no difference between the knowledge of the providers.

### Educational qualification impact on VAP prevention practices

Higher education correlates positively with adherence to VAP bundle with (*p* < 0.05). Similarly, Education appeared as a crucial factor influencing various medical procedures to prevent ventilator associated pneumonia. Specifically, the use of subglottic endotracheal tubes, selection of kinetic patient beds, and the use of 0.12% chlorhexidine gluconate antiseptic oral rinse were all significantly associated with educational categories. This aligns with several studies conducted by that higher educational degrees may enhance these practices and reduce the incidence of VAP (27, 28).

### Professional experience and practical skills

Professional experience proven that less experienced individuals show better adherence. While strong associations were found with advanced analysis with all the medical procedures and practices examined in the study, emphasizing the significance of practical skills and knowledge accumulated over time. This suggests that mentorship programs or practical training could be beneficial for less experienced professionals. Interestingly, this aligned with another multiple studies that found clear association of knowledge between experienced and less experienced nurses (25, 29, 30).

### Age and nationality

Increasing age shows a positive correlation with adherence to the bundle. Additionally, nationality shows potential differences in adherence with various medical procedures. For example, the type of airway humidifier was significantly associated with age while not with nationality. These associations may be attributed to cultural differences or age-related changes in practice, emphasizing the need for further research to understand these better. However, the research team did not find recent studies to investigate the relationship between the age and nationality of the healthcare workers and the VAP bundle knowledge. Therefore, examining the correlation between both variables is required and crucial to be investigated.

### Training courses and adherence to VAP bundle

The findings of this study were consistent with several studies indicating that lack of education and training was consistently identified as the primary reason precluding proper implementation of the VAP bundle. EBP training courses on VAP were associated with several questions, including the choice between oral and nasal routes for endotracheal intubation, usage of subglottic endotracheal tubes, selection of kinetic patient beds, use of 0.12% chlorhexidine gluconate antiseptic oral rinse, and performance of the spontaneous breathing trial *p* < 0.05. This underscores the substantial impact of training courses on these practices and needed further investigation (21, 29–33). In conclusion, this analysis offers valuable insights into the factors influencing the comprehension and adherence of critical care health professionals to the VAP bundle. The study not only shows significant associations but also highlights areas for further exploration and intervention, contributing to the ongoing efforts to enhance patient care and safety in critical care settings.

## Recommendations

Ventilator associated pneumonia poses a significant threat to patients in critical care, leading to heightened morbidity, mortality, and healthcare costs. This study proposes several recommendations based on its findings to bolster VAP prevention and enhance patient outcomes. The paper aims to succinctly outline these recommendations for future implementation and research. Critical care practitioners often grapple with resource limitations, such as insufficient staffing and restricted access to necessary supplies, impeding their efforts in VAP prevention. Our study finding agrees with several studies ongoing education programs for all healthcare workers are imperative to ensure adherence to evidence-based practices, emphasizing the need for effective communication and collaboration among them. Leaders play a pivotal role in promoting a culture of VAP prevention, and further research is warranted to explore additional factors influencing prevention efforts. The recommendations include addressing resource limitations by prioritizing sufficient resources, implementing education programs focusing on evidence-based practices, promoting communication and collaboration through interdisciplinary meetings, fostering a culture of VAP prevention led by nurse leaders, and conducting further research to explore additional influencing factors. By adhering to these recommendations, healthcare facilities can bolster VAP prevention, enhance patient outcomes, and create a safer environment for critically ill patients, emphasizing the importance of gender-specific factors, education and training opportunities, professional experience, mentorship, and demographic considerations in intervention design. It is imperative for healthcare institutions and stakeholders to prioritize and implement these recommendations effectively to prevent VAP and optimize patient care in critical care settings.

## Conclusion

Ventilator-associated pneumonia is a prevalent nosocomial infection, affecting 10–70% of intensive care unit patients and contributing to prolonged hospital stays at a rate of 20–30%. Incidence within the ICU rises to 22.8% among mechanically ventilated patients, comprising 47% of all ICU infections and impacting 9–27% of intubated individuals. VAP significantly extends ICU and hospital durations, elevating healthcare costs and fostering antimicrobial resistance. To combat this, Healthcare institutions develop the Ventilator-Associated Pneumonia prevention bundle, consisting of five key interventions. While this bundle has been extensively examined, there is a need for research comparing knowledge and practices of specialists in tertiary hospitals in Riyadh, addressing knowledge gaps and promoting practices to mitigate adverse outcomes. This study underscores the role of education, professional experience, and demographics in shaping medical procedures and practices, suggesting targeted interventions could enhance bundle adherence.

This research identifies key factors influencing VAP prevention in critical care settings and offers actionable recommendations for healthcare institutions to enhance patient safety. Challenges faced by critical care professionals emphasize the importance of targeted interventions like addressing resource limitations, particularly staffing issues. Adequate staffing ensures consistent implementation of the VAP bundle. Education’s pivotal role in influencing healthcare practices is reinforced, emphasizing ongoing programs tailored to critical care practitioners’ needs. Fostering a culture of prevention, supported by healthcare leaders, is essential for embedding these practices into daily routines. Multidisciplinary collaboration is crucial, and tailoring interventions based on demographic factors can enhance their impact. Continuous research and quality improvement initiatives are essential for evolving best practices. In conclusion, addressing resource challenges, prioritizing education, fostering a culture of prevention, and promoting interdisciplinary collaboration can create an environment conducive to consistent adherence to VAP prevention practices, advancing patient safety in critical care settings.

## Data Availability

The raw data supporting the conclusions of this article will be shared upon reasonable request and approval.
